# Comment on: Resistance profiles of carbapenemase-producing Enterobacterales in a large centre in England: are we already losing cefiderocol?

**DOI:** 10.1093/jac/dkaf074

**Published:** 2025-03-12

**Authors:** Christopher M Longshaw, Boudewijn L M Dejonge, Yoshinori Yamano

**Affiliations:** Medical Affairs, Shionogi B.V., London, UK; Medical Affairs, Shionogi Inc., Florham Park, NJ, USA; Laboratory for Drug Discovery and Disease Research, Shionogi & Co., Ltd, Osaka, Japan

We were concerned to read the recent research paper by Baltas *et al*.^1^ ‘*Resistance profiles of carbapenemase-producing Enterobacterales in a large centre in England: are we already losing cefiderocol?*’ describing apparently high levels of resistance to cefiderocol in isolates of NDM-producing Enterobacterales from their institution.

While resistance levels can vary locally, these results are in stark contrast to longitudinal surveillance studies that have reported consistently low levels of background resistance to cefiderocol in Europe and the USA.^2^ According to the most recent data presented at IDweek 2024, 62.1% of NDM-1-carrying carbapenem non-susceptible Enterobacterales collected in Europe and the USA from 2020 to 2023 were susceptible at the EUCAST breakpoint of 2 mg/L but when interpreted by the less conservative CLSI/FDA breakpoint of 4 mg/L,^3^ 91.4% of the isolates were susceptible. These percentages are markedly higher than the 12.1% susceptibility reported by Baltas.

The discrepancy may be better understood as Baltas *et al*. only employed the disc diffusion method and did not confirm resistance by the reference broth microdilution method using iron-depleted cation-adjusted Mueller Hinton broth.

Examination of zone diameter distributions in Figure 2b from Baltas *et al.* (redrawn here as Figure 1a) highlights most NDM-producing isolates had zone diameters of <23 mm, the current EUCAST breakpoint for Enterobacterales, with the majority having zone diameters of <21 mm and 24% falling in the area of technical uncertainty (ATU) between 21 and 23 mm. It’s worth noting that applying the zone diameter breakpoints prior to Jan 2024 (EUCAST v13) results in 33/66 (50%) isolates falling within the ATU (Figure 1b), and if one interprets the same data using the CLSI/FDA disk diffusion breakpoints 52/66 (79%) of these isolates would have been reported susceptible to cefiderocol (Figure 1c).

**Figure 1.**
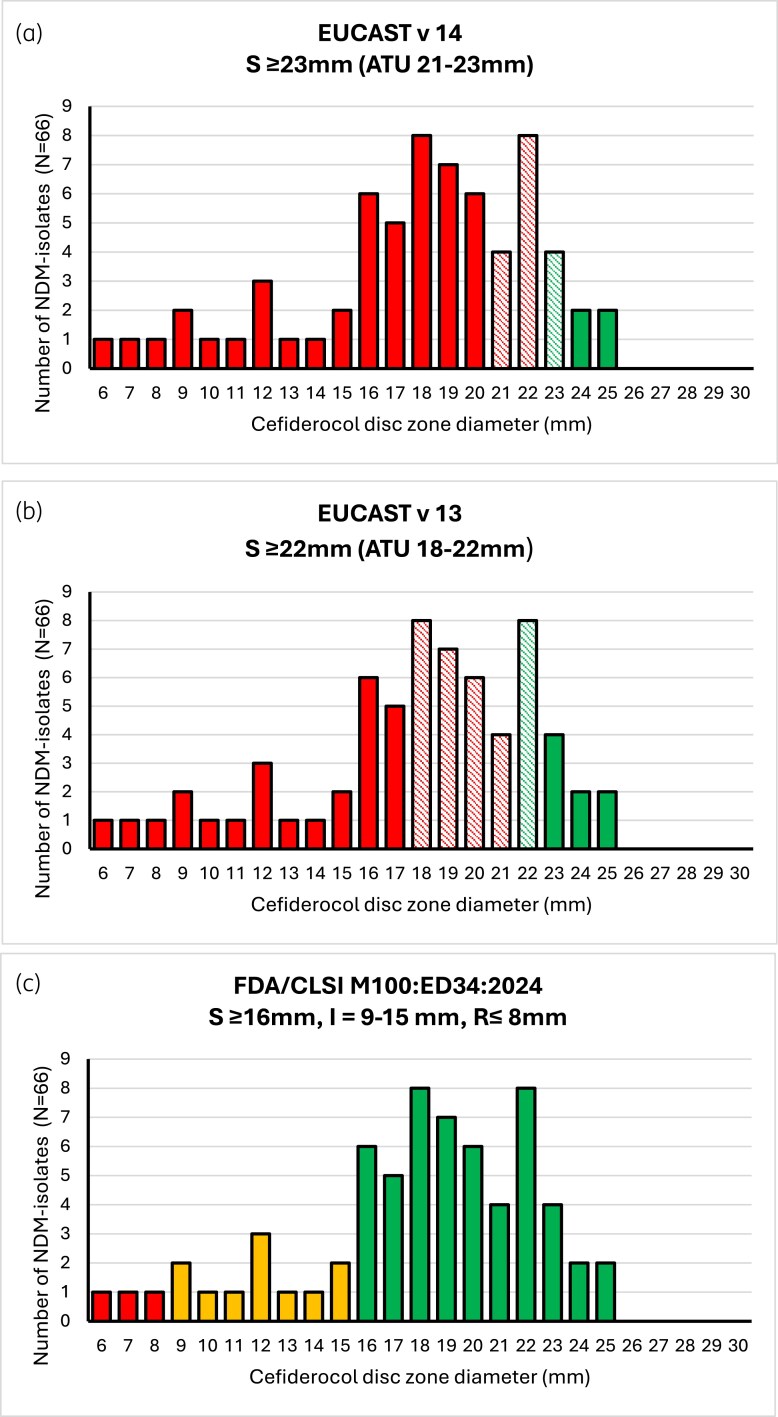
Comparison of cefiderocol disc zone diameter results for NDM-producing and NDM/OXA-48-producing isolates (*n* = 46) from Baltas *et al*.^1^ interpreted by (a) 2024 EUCAST Breakpoints version 14.0; (b) 2023 EUCAST Breakpoints version 13.0; (c) 2024 FDA/CLSI M100 ED34:2024. Green: susceptible; amber: intermediate; red: resistant; hatched lines denote area of technical uncertainty.

The discrepancy between EUCAST and FDA/CLSI breakpoints was highlighted by the authors, predicting that the variation would lead to different use of cefiderocol between countries based on which breakpoint recommendations they follow.

In 2024, EUCAST narrowed the ATU to 21–23 mm in an effort to reduce ambiguous reporting and improve performance. Unfortunately, whilst the number of isolates falling into the ATU has been reduced, this revision has also led to an increase in the number of false resistant isolates being reported.

In the 2022 EUCAST disc correlation study, Matsuchek *et al.*^4^ cautioned ‘…*the advice for isolates that fall within the ATU is that they should be retested by a different method (i.e. broth microdilution), reported as resistant or not reported at all. Without re-resting, there is a risk that potentially susceptible isolates with MIC* ≤*2 mg/L but zone diameters in the 18–22 mm range are wrongly classed as resistant, which has potential negative implications for patient care due to the limited treatment options…’*.

A number of independent published studies^5–8^ have since confirmed that high percentages of isolates either reported in the ATU or resistant by disk were actually susceptible with MIC ≤2 mg/L when tested by broth microdilution using ID-CAMHB and recommended such isolates should be routinely retested by broth microdilution to truly assess their susceptibility.

There are currently two commercially available broth microdilution-based tests for cefiderocol: UMIC^®^ Cefiderocol (Bruker Daltonics GmbH & Co.) and ComASP^®^ Cefiderocol 0.08–128 (Liofilchem srl), both of which use ID-CAMHB and are based on the reference ISO 20776-1:2019 method. Both tests have high levels of reproducibility (>95%)^9,10^ and are approved and CE-marked in Europe according to IVDD or IVDR requirements. ComASP^®^ also gained clearance in the USA under the FDA 510K requirements.

The potential for misinformation from epidemiological reporting of resistance levels based solely on disc diffusion is also illustrated by another recent paper, in which Ljungquist and collaborators reported alarmingly high levels of cefiderocol resistance, including pan-drug resistance in isolates of *Klebsiella pneumoniae* recovered from victims of the ongoing conflict in Ukraine being treated in Sweden.^11^ In that study, the reported cefiderocol resistance percentage of 81% using EUCAST v14 breakpoint criteria is reduced to 16.2% when interpreted according to EUCAST v13 and only 2.7% of isolates are resistant by CLSI/FDA breakpoint criteria. The clinical implications of the different breakpoint interpretations are stark, as patients with limited therapeutic options who could receive cefiderocol as appropriate therapy in the USA would most likely be denied this option if admitted to a hospital in Europe.

The responsible use of reserve antibiotics is critical when faced with increasing global antimicrobial resistance, and determination of local resistance is paramount for good stewardship. Care needs to be taken, however, to ensure reported resistance reflects the true risk of treatment failure and is not exaggerated as a consequence of cautious breakpoint criteria and use of non-reference methods so prescribers can be confident that all available treatment options are considered for their patients.

## References

[1] Baltas I , Patel T, Soares AL. Resistance profiles of carbapenemase-producing Enterobacterales in a large centre in England: are we already losing cefiderocol? J Antimicrob Chemother 2025; 80: 59–67. 10.1093/jac/dkae367 39504496 PMC11695913

[2] Wise MG , Karlowsky JA, Hackel MA et al In vitro activity of cefiderocol against meropenem-nonsusceptible gram-negative bacilli with defined β-lactamase carriage: SIDERO-WT surveillance studies, 2014–2019. Microb Drug Resist 2023; 29: 360–70. 10.1089/mdr.2022.0279 37253158 PMC10387160

[3] Mendes RE , Kimbrough H, Beekman D et al P-1363. Cefiderocol activity against clinical Enterobacterales isolates carrying metallo-ß-lactamase genes in United States and European Hospitals (2020–2023). Open Forum Infectious Diseases 2025; 12 Suppl 1: S848. 10.1093/ofid/ofae631.1540

[4] Matuschek E , Longshaw C, Takemura M et al Cefiderocol: EUCAST criteria for disc diffusion and broth microdilution for antimicrobial susceptibility testing. J Antimicrob Chemother 2022; 77: 1662–9. 10.1093/jac/dkac080 35289853 PMC9155621

[5] Bianco G , Boattini M, Comini S et al Disc diffusion and ComASP^®^ cefiderocol microdilution panel to overcome the challenge of cefiderocol susceptibility testing in clinical laboratory routine. Antibiotics (Basel) 2023; 12: 604. 10.3390/antibiotics12030604 36978470 PMC10045311

[6] Bianco G , Boattini M, Comini S et al Performance evaluation of bruker UMIC^®^ microdilution panel and disc diffusion to determine cefiderocol susceptibility in Enterobacterales, Acinetobacter baumannii, Pseudomonas aeruginosa, Stenotrophomonas maltophilia, Achromobacter xylosoxidans and Burkolderia species. Eur J Clin Microbiol Infect Dis 2024; 43: 559–66. 10.1007/s10096-024-04745-7 38240988

[7] Castillo-Polo JA , Hernández-García M, Maruri-Aransolo A et al Cefiderocol AST in a real-life *Klebsiella pneumoniae* collection: challenges in the ATU range. J Antimicrob Chemother 2025; 80: 797–801. 10.1093/jac/dkae477 39811880

[8] Stracquadanio S , Nicolosi A, Marino A et al Issues with cefiderocol testing: comparing commercial methods to broth microdilution in iron-depleted medium—analyses of the performances, ATU, and trailing effect according to EUCAST initial and revised interpretation criteria. Diagnostics (Basel) 2024; 14: 2318. 10.3390/diagnostics14202318 39451641 PMC11506871

[9] Dortet L , Niccolai C, Pfennigwerth N et al Performance evaluation of the UMIC^®^ cefiderocol to determine MIC in gram-negative bacteria. J Antimicrob Chemother 2023; 78: 1672–6. 10.1093/jac/dkad149 37209112 PMC10320108

[10] Koeth LM , DiFranco-Fisher JM, Palavecino E et al A multicenter performance evaluation of cefiderocol MIC results: ComASP in comparison to CLSI broth microdilution. J Clin Microbiol 2025; 63: e0092624. 10.1128/jcm.00926-24 39745470 PMC11837567

[11] Ljungquist O , Magda M, Giske CG et al Pandrug-resistant Klebsiella pneumoniae isolated from Ukrainian war victims are hypervirulent. J Infect 2024; 89: 106312. 10.1016/j.jinf.2024.106312 39396555

